# Social context affects sequence modification learning in birdsong

**DOI:** 10.3389/fpsyg.2025.1488762

**Published:** 2025-02-05

**Authors:** Lioba Fortkord, Lena Veit

**Affiliations:** Neurobiology of Vocal Communication, Institute for Neurobiology, University of Tübingen, Tübingen, Germany

**Keywords:** songbird, vocalization, social context, motor sequencing, learning

## Abstract

Social interactions are crucial for imitative vocal learning such as human speech learning or song learning in songbirds. Recently, introducing specific learned modifications into adult song by experimenter-controlled reinforcement learning has emerged as a key protocol to study aspects of vocal learning in songbirds. This form of adult plasticity does not require conspecifics as a model for imitation or to provide social feedback on song performance. We therefore hypothesized that social interactions are irrelevant to, or even inhibit, song modification learning. We tested whether social context affects song sequence learning in adult male Bengalese finches (*Lonchura striata domestica*). We targeted specific syllable sequences in adult birds’ songs with negative auditory feedback, which led the birds to reduce the targeted syllable sequence in favor of alternate sequences. Changes were apparent in catch trials without feedback, indicating a learning process. Each experiment was repeated within subjects with three different social contexts (male–male, MM; male–female, MF; and male alone, MA) in randomized order. We found robust learning in all three social contexts, with a nonsignificant trend toward facilitated learning with social company (MF, MM) compared to the single-housed (MA) condition. This effect could not be explained by the order of social contexts, nor by different singing rates across contexts. Our results demonstrate that social context can influence degree of learning in adult birds even in experimenter-controlled reinforcement learning tasks, and therefore suggest that social interactions might facilitate song plasticity beyond their known role for imitation and social feedback.

## Introduction

Humans learn language by imitative vocal learning, making social interactions with conspecifics crucial for speech and language acquisition (Kuhl, 2004; Goldstein and Schwade, 2008). Songbirds are an important animal model for vocal learning, as they also acquire their song through vocal imitation, with many well-established parallels to human language learning (Doupe and Kuhl, 1999). Juvenile birds learn to imitate an adult tutor heard early in life. Tutoring is also possible with playback of tutor stimuli from a speaker, but it is enhanced by social interaction with live tutors or interactive components of the tutoring apparatus (Catchpole and Slater, 2008; Deregnaucourt et al., 2013; Chen et al., 2016; Varkevisser et al., 2022). In species in which only male birds sing, feedback on song performance from non-singing females can also enhance the learning process (West and King, 1988; Carouso-Peck and Goldstein, 2019; Roeser et al., 2023; Bistere et al., 2024).

In contrast, social interactions may be irrelevant or even inhibit a form of song plasticity in which experimenter-controlled modifications are introduced into adult song via differential reinforcement. (Tumer and Brainard, 2007; Andalman and Fee, 2009; Warren et al., 2012; Ali et al., 2013; Zai et al., 2020; McGregor et al., 2022; Tachibana et al., 2022a; Kawaji et al., 2024). For example, birds learn to adaptively modify their songs to avoid negative auditory feedback in the form of a short burst of white noise (Tumer and Brainard, 2007; Warren et al., 2012). These reinforcement learning protocols have emerged as a key tool to study the song learning process, because they allow modification of specific song features, such as syllable structure or sequencing, in adult birds. Learned song changes rely in part on the same neuronal circuit which is also involved in juvenile song learning (Andalman and Fee, 2009; Warren et al., 2011; Tian and Brainard, 2017; Hisey et al., 2018; Xiao et al., 2018). This learning circuit is more active during undirected singing and suppressed when adult birds perform female-directed courtship song (Kao et al., 2005; Sakata and Brainard, 2009; Kojima and Doupe, 2011; Woolley et al., 2014; Singh Alvarado et al., 2021), consistent with a switch from undirected ‘practice’ to female-directed ‘performance’ song (Jarvis et al., 1998; Singh Alvarado et al., 2021). We therefore hypothesized that these socially-driven circuit changes may prevent song modification learning when subjects are co-housed with other birds. Since reinforcement learning protocols have only been tested in single-housed birds, it is unknown whether the presence of conspecifics inhibits, or, conversely, facilitates this form of non-imitative vocal learning.

Here, we study the effect of social context on song sequence learning in adult Bengalese finches (*Lonchura striata domestica*). Bengalese finch song is composed of variable sequences of distinct syllables and is ideally suited to investigate the composition of complex behaviors that are organized in syntactical sequences (Lashley, 1951; Okanoya, 2004; Berwick et al., 2011; Lipkind et al., 2013). The relative transition probabilities between syllables are typically stable, but birds can learn to modify probabilities of specific transitions in response to differential reinforcement (Warren et al., 2012; Veit et al., 2021). We found that male Bengalese finches show robust sequence learning in the presence of male and female conspecifics, and that degree of learning in social conditions can even be enhanced over the single-housed condition.

## Methods

### Subjects

Experiments were carried out on six adult male Bengalese finches (*Lonchura striata domestica*) from the lab’s breeding colony (mean age ~333 days post hatch (dph), range 181–488 dph). All birds were housed in same-sex social groups in large indoor aviaries and were familiar with opposite-sex birds through visual and acoustic contact in neighboring aviaries. All experiments were performed in accordance with animal protocols approved by the national authority, the Regierungspräsidium Tübingen, Germany.

### Experimental timeline

A prescreening procedure with a social partner of each sex ensured that each male subject sang enough in each context. If the singing rate was strongly suppressed, we repeated the prescreening with a different social partner. We had to screen an average of 1.8 partners (range 1–3) for the MM and 1.2 partners (range 1–2) for the MF condition.

**Figure 1:**
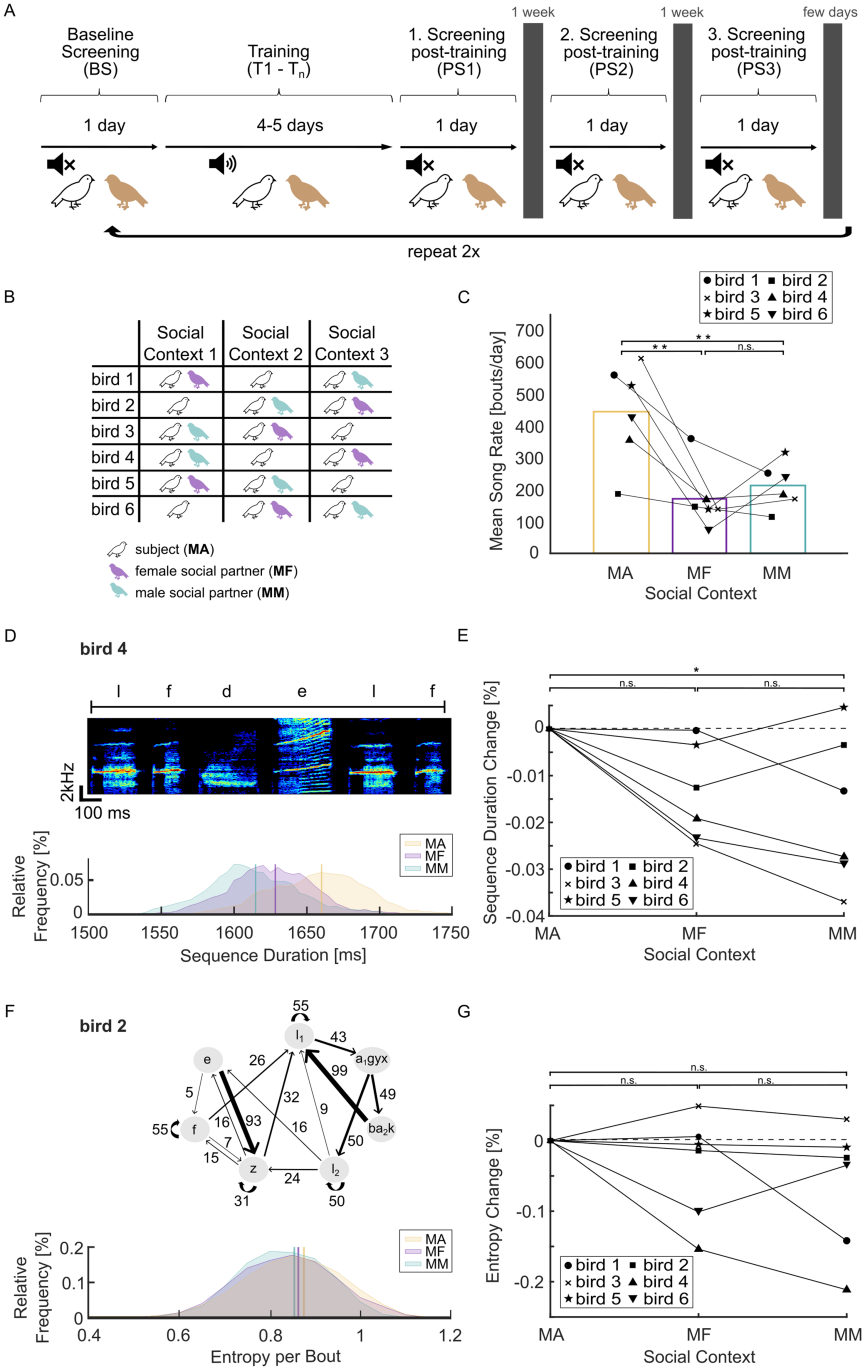
Social context affects song rate and song speed. **(A)** Schematic of the training protocol. After progressing through all stages of training and screening for one social context, the protocol is repeated for the other social contexts. White-noise (WN) training is indicated by the speaker symbol. **(B)** Order of social contexts for each male subject. **(C)** Mean song rate for each social context (MA = male alone, MF = male–female, MM = male–male). **p < 0.01, multiple comparison test following one-way ANOVA. **(D)** Example data of bird 4. Top: Example spectrogram of the syllable sequence used for determining song speed during BS (baseline screening). Letters mark individual syllables. Bottom: Distribution of sequence durations for this syllable sequence for each social context (binsize = 5; kernel used for smoothing = 6). Vertical bars indicate mean sequence duration (MA, 1,660 ms; MF, 1,628 ms; MM, 1,615 ms). **(E)** Average change in song speed relative to the MA context on a group level. *p = 0.038, multiple comparison test following one-way ANOVA. **(F)** Example data of bird 2. Top: Transition diagram showing all possible transitions in the repertoire of the example bird. Numbers on edges show transition probabilities in %, letters in the nodes denote syllables and chunks (stereotyped syllable sequences). Subscripts indicate different states of acoustically the same syllable type, defined by their sequential context as in Koparkar et al. (2024). Transitions from a syllable or chunk may not sum to 100% due to omitting transitions <5% for clarity. Bottom: transition entropy per bout (bin size = 0.05; kernel used for smoothing = 6). Vertical bars indicate mean entropy (MA, 0.88; MF, 0.86; MM, 0.85). **(G)** Average change in transition entropy relative to the MA context. There was no significant difference in entropy between any of the social contexts on a group level (one-way ANOVA, factor ‘social context’: *p* = 0.29).

At the start of the experiment, the subject was acclimatized to the setup for 24 h. After that, the following periods were completed for one social context (Figure 1A): 24 h of baseline screening (BS); training with negative auditory feedback for 4–5 days (T1–T5); 24 h of post-screening without WN (post screening, PS 1). The subject was then returned to the aviary and screening was repeated in the same social context approximately 1 and 2 weeks after the end of training (PS 2 and 3), to observe the return to baseline. After PS3 and return to the aviary, BS started for the next social context (Figure 1A). Each male subject experienced training on the same branch point in three social contexts (male–male, MM; male–female, MF; male alone, MA). The order of contexts was randomized and balanced (Figure 1B).

### Experimental procedure

The subjects were situated in experimental cages (120 × 50 × 50 cm) in sound-attenuating boxes equipped with dimming 14:10 light cycles and several forms of enrichment. Sound was recorded using the custom LabView software EvTAF (Tumer and Brainard, 2007; Veit et al., 2021) with a microphone (Rode, Sydney, Australia) and an analog amplifier (RME, Haimhausen, Germany). A branch point is a syllable that can be followed by multiple other syllables. For each bird, one branch of a branch point was targeted with negative auditory feedback to reduce transition probability to the target branch. EvTAF was used for real-time delivery of short bursts of white noise (WN, 40 ms). WN was omitted from 10% of recordings (catch trials) to quantify learning.

### Data analysis

Proportion tests were performed in R (v4.4.1; R Core Team, 2024). All remaining data analysis and statistical tests were performed with MATLAB R2023b and the Statistics and Machine Learning toolbox (MATLAB, 2023).

### Targeting accuracy

To control for targeting accuracy, we calculated correct hits, false positives and misses of WN. The average template accuracy across all training days was 92.78%. We excluded a training day with targeting accuracy lower than 75% from analysis (T5_MF in bird 1).

### Syllable sequence annotation

We performed song sequencing and syllable annotation separately for each subject, as in Koparkar et al. (2024): A song bout was defined as a continuous period of song separated by 2 s of silence. A subset of bouts was used for unsupervised syllable clustering after UMAP projection (McInnes et al., 2020; Sainburg et al., 2020). Clusters were manually verified and corrected before training a deep neural network, TweetyNet, to annotate remaining songs (Cohen et al., 2022). Annotations were semi-manually checked and corrected using custom-written MATLAB code. For the MM context, we manually separated bouts which contained only the subject’s song from bouts containing song of both male birds or only the social partner. If song bout files contained mixed song, we excluded these files if there was substantial temporal overlap between the two males (e.g., 0.94% of files, range 0–3.37% for the first day of training; 4.19% of files, range 0–8.82% for the last day of training).

### Learning

**Figure 2:**
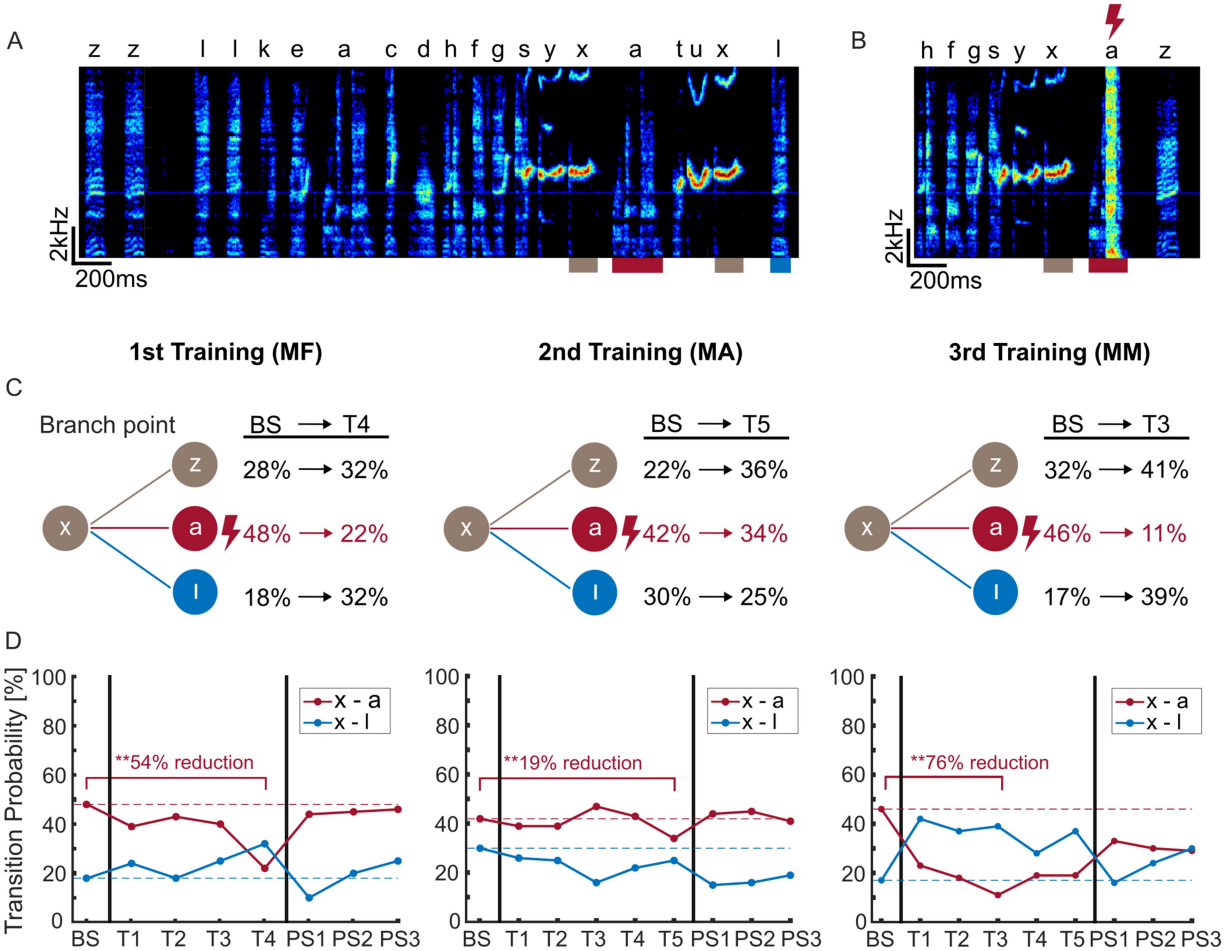
Example sequence learning data for bird 1. **(A)** Example spectrogram of a song bout. The branchpoint syllable ‘x’ is marked in brown and two alternative successive syllables ‘a’ and ‘l’ are indicated in red and blue. **(B)** Example spectrogram section during WN-training, in which syllable ‘a’ after ‘x’ was targeted with WN. The WN appears as a bright line in the spectrogram, marked by a lightning bolt. **(C)** Transition probabilities for the different branches during baseline screening (BS) and the best day of training for all social contexts in the order presented to bird 1 (from left to right: MF = male–female, MA = male alone, MM = male–male). Branchpoint syllable ‘x’ had two main alternative branches, ‘a’ and ‘l’. The third branch, syllable ‘z’ includes all possible other transitions from syllable ‘x’, including to the end of bouts. **(D)** Learning curves in all social contexts, order as in panel **(C)**. Transition probabilities are shown for the targeted transition ‘x – a’ and one alternative transition ‘x – l’ for BS (baseline screening), training days (T1–T4/5) and post-screenings (PS1–PS3). Dashed lines indicate baseline. The degree of learning (reduction of the targeted transition) is noted above the red bar.

To quantify the degree of learning, we determined transition probabilities (TPs) to the targeted branch and alternative non-target branch(es) following the branch point syllable (Figure 2C). We computed the degree of learning, using only catch trials, as follows:


$$Learning\left[\%\right]=100-\left(\frac{\begin{array}{l} TPto\;thetargeted\;branchonthe\\ \;bestdayof\;training \end{array}}{\begin{array}{l} TPto\;the\;targeted\;branch\;\\ during\;baseline\;screening \end{array}}\cdot 100\right)$$

**Figure 3:**
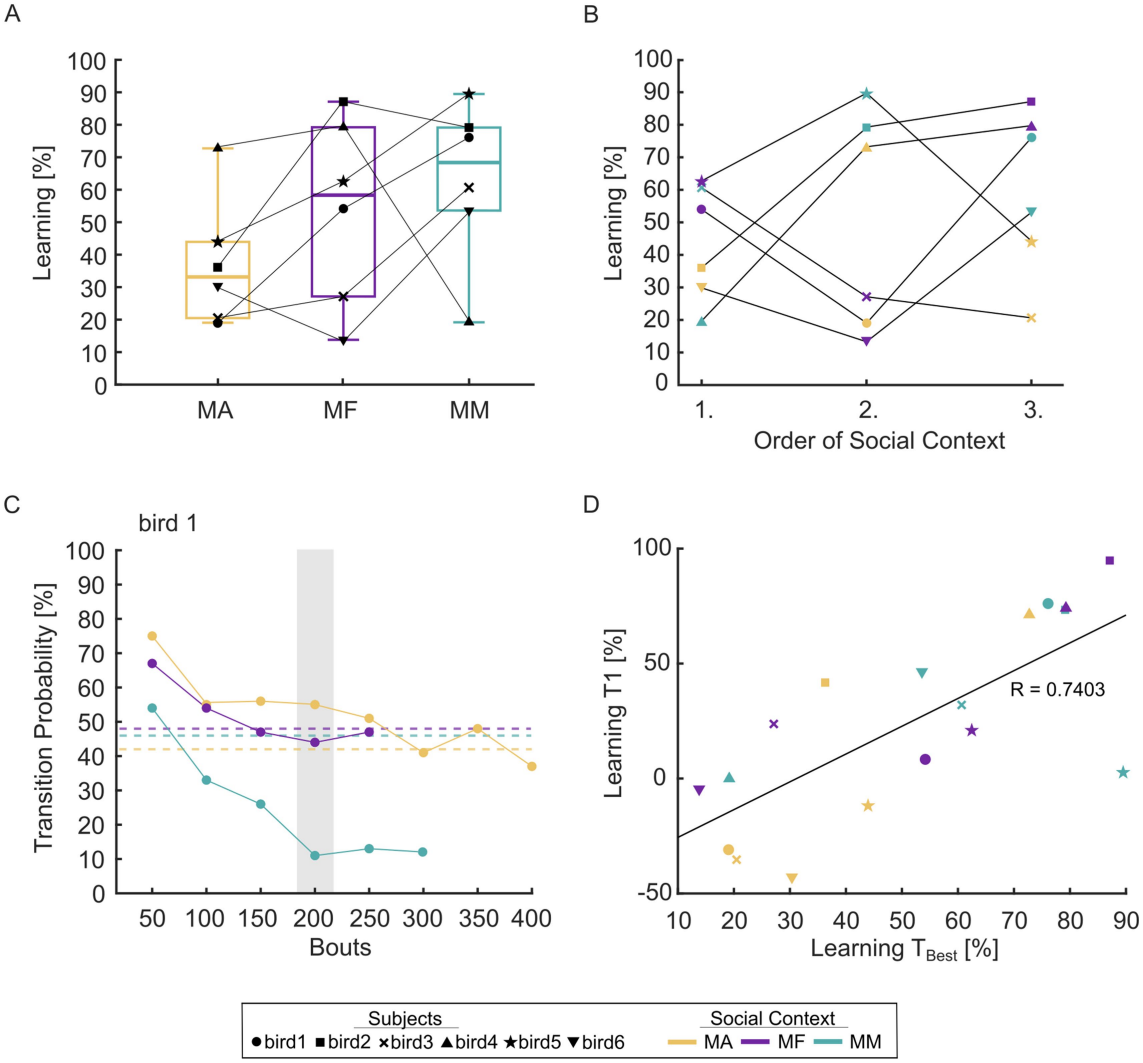
Degree of learning as a factor of social context. **(A)** Total degree of learning [% reduction against baseline] as a factor of social context. Boxes indicate the lower and upper interquartile range, line indicates median. Whiskers indicate the minimum and maximum data values. **(B)** Degree of learning [% reduction against baseline] as a factor of training order. **(C)** Example learning curves within the first training day for bird 1. Transition probabilities were determined in bins of 50 bouts. To quantify degree of learning at the end of the first training day, the last complete bin of the social context with the lowest song rate was chosen and the same bin was used for all remaining social contexts (e.g., bin 150–200 for bird 1, marked by the shaded box). Dashed lines indicate transition probability during baseline screening in each context. **(D)** Correlation between degree of learning at the end of the first training day and total degree of learning (as in Figure 3A). Colors indicate social context (MA, male alone; MF, male–female; MM, male–male). The individual birds are indicated by different marker styles.

To specifically capture differences in the time course of early learning, we additionally computed TPs on the first training day with a bin size of 50 bouts. To approximate learning at the end of T1 (for Figure 3D), we chose the last complete bin of the social context with the lowest song rate for each subject (marked by a shaded box for the example bird in Figure 3C) and used the same bin for all remaining social contexts. The relative speed of learning was defined as the degree of learning divided by total song rate.

### Song rate and speed

Song rate on full days of BS and training was quantified as the number of recorded bouts between 9:30 a.m. and 5 p.m. To measure song speed, we chose a syllable sequence which occurred in the same order [“chunk,” (Seki et al., 2008)] and calculated its duration from the onset of the first syllable to the offset of the last syllable during BS. This measure of sequence duration thus includes both syllable and gap duration changes across social contexts.

### Transition entropy

Transition entropy is a measure of sequencing variability. We determined the average transition entropy per bout during BS as follows (Sakata and Brainard, 2009; Koparkar et al., 2024): The entropy *H_a_* of syllable ‘a’ is calculated by computing *P(i),* the probability of the *i*th outcome, with *n* number of different outcomes.


$$H_{a}=-\overset{n}{\underset{i=1}{\sum }}P\left(i\right)logP\left(i\right)$$

The total transition entropy *TE* of song was calculated over all syllables *b*. *H_b_* is the transition entropy at *b* and *P(b)* is the frequency of syllable *b*.


$$TE=\overset{n}{\underset{b=1}{\sum }}H_{b}*P\left(b\right)$$

## Results

### Singing rate is reduced in social conditions

We tested how song features and degree of learning varied across social contexts in six adult male Bengalese finches. Each bird experienced three different social contexts (male alone, MA; male–female, MF; male–male, MM) in randomized order (Figures 1A,B). Female-directed song is typically studied by presenting a novel female for a brief time (female exposure typically <2 min: Sakata and Brainard, 2009; Aronov and Fee, 2012; Picardo et al., 2016; Toccalino et al., 2016) and typical female-directed song changes attenuate after a longer period of co-housing (Heinig et al., 2014, within 1 h). We therefore speculated that differences in baseline song might be negligible during longer co-housing in our experiment. We found that average singing rate during baseline screening (BS) was significantly reduced in social conditions compared to the single-housed condition (one-way ANOVA, factor social context: *F*_2,15_ = 9.97, *p* = 0.0018, n = 6 birds; Multiple Comparison: MA-MF, *p* = 0.0023; MA-MM, *p* = 0.0083; MF-MM, *p* = 0.80; Figure 1C), consistent with previous results (Yamahachi et al., 2017). Qualitatively the same results were obtained for maximum singing rate in shorter time intervals (Supplementary Figure 3).

### Song speed is affected by social context

Female-directed song is typically faster than undirected song (Sakata et al., 2008; Aronov and Fee, 2012), and little is known about male-directed song in zebra finches or Bengalese finches (Chen et al., 2016). We measured song speed using the duration of a fixed syllable sequence. The example bird (bird 4) in Figure 1D showed increased song speed in the social conditions compared to the MA context. Overall, there was a significant effect of social context on song speed (one-way ANOVA, social context: *F*_2,15_ = 4.2, *p* = 0.036, n = 6 birds; Multiple comparison: MA-MF, *p* = 0.11; MA-MM, *p* = 0.038; MF-MM, *p* = 0.84) (Figures 1D,E) even during prolonged co-housing with conspecifics in our experiments.

### Sequence variability is not affected by social context in our sample

Previous studies show that female-directed song of Bengalese finches is accompanied by a decrease in transition entropy, a measure for variability in syllable sequencing (Sakata et al., 2008). Here, we did not find a consistent change in total transition entropy (one-way ANOVA, social context: *F*_2,15_ = 1.35, *p* = 0.29, *n* = 6 birds; Figures 1F,G). These average daily values might represent a mixture of directed and undirected song bouts. We therefore investigated song speed and transition entropy values on a bout-by-bout level but were unable to distinguish directed from undirected singing using these values in either the MF or MM social contexts (Supplementary Figure 1). Overall, we found that social context affected song rate and song speed, but not transition entropy, in a way that persisted during co-housing in the same experimental cage for extended periods.

### Sequence learning occurs in all three social contexts

Figures 2A,B show example spectrograms (bird 1) with the branch point syllable (‘x’) and the following syllables (target syllable ‘a’ and alternative syllables ‘l’ & ‘z’). By targeting a syllable with a short burst of white noise (WN) (Figure 2B), birds reduce transition probabilities to the target syllable in favor of alternative syllable(s) on subsequent bouts (Warren et al., 2012; Veit et al., 2021). This behavior represents a learning process that is apparent also in catch trials without WN feedback. Learning curves in Figure 2D show the transition probability to the target syllable over 4–5 days of training. In this example bird, we found the largest reductions in transition probabilities to the target syllable in the MF and MM social contexts (reductions: MF 54%, MM 76%), in comparison to the MA social context (reduction: 19%; Figures 2C,D). In all social contexts, transition probability to the target sequence was significantly reduced after training (*p* < 0.001 for all birds except bird5_MF (*p* = 0.5), proportion test, Supplementary Table 1). The average reduction was 38.8% in the MA context, 46.0% in the MF context, 63.6% in the MM context. We concluded that learning is possible in all social contexts.

### Learning is typically enhanced in the social conditions

For five out of six birds, degree of learning was higher in the MM context compared to the MA context (Figure 3A). We did not find a significant main effect of social context on degree of learning at the group level (3-way ANOVA, social context, order, subject: *F*_*2*,8_ = 1.64, *p* = 0.25). However, on average, learning in the MM condition tended to be 63.8% higher than learning in the MA condition.

Since the degree of learning tended to be higher in the social conditions, in which singing rate was reduced (Figure 1C; Supplementary Figure 2), we wondered whether the differences between social contexts would be more apparent in the song-by-song dynamics of sequence changes. We therefore plotted transition probabilities in bins of 50 bouts only within the first day of training. A faster and larger decrease in transition probability to the target syllable was often observed in one of the social conditions (Figure 3C), and the relative speed of learning was significantly increased in social conditions compared to the MA condition (paired t-tests: *p* = 0.022 MA-MF, *p =* 0.048 MA-MM, *n* = 6 birds). The learning progress measured in the same 50-bout bin on the first training day was predictive of the total degree of learning (Figure 3D, correlation: *r* (16) = 0.7403, *p* = 0.0004).

### No effect of training order

Veit et al. (2021) showed that repeated sequence training in rapidly alternating contexts eventually led birds to remember context-specific changes. Moving from the aviary into the experimental cage could represent one such context change which birds could use to immediately shift sequencing. Here, we accounted for this possibility by assigning the social contexts in a random and balanced order across all birds. Overall, we did not find a significant main effect of training order (3-way ANOVA, social context, training order, subject: *F*_2,8_ = 0.59, *p* = 0.57). We could not observe a common pattern across birds (Figure 3B), suggesting that three exposures are likely not enough to form context-dependent motor savings (Veit et al., 2021).

## Discussion

Songbirds learn their species-specific song through vocal imitation, a process which is enhanced by social interactions with live conspecifics. It was unknown whether the social environment can also influence learning in experimenter-controlled reinforcement learning tasks, in which adult birds learn to change a specific feature of their song in response to negative auditory feedback. We used a within-subject design to test the influence of social context on Bengalese finches’ degree of learning during sequence modification learning. We found no significant main effect of training order or social context, but a trend toward stronger and faster learning in the social conditions (male–male, MM, or male–female, MF) compared to the single-housed condition (male alone, MA). While these data come from relatively few animals, they demonstrate that sequence modification learning is possible in social housing and can even be facilitated by the presence of a social partner.

### Social influences on song performance

The presence of male or female conspecifics has well-described influences on birdsong performance, such as during female-directed courtship song (Sossinka and Böhner, 1980; Sakata et al., 2008) or territorial countersinging interactions (Searcy and Beecher, 2009; King and McGregor, 2016; Alcami et al., 2021; Costalunga et al., 2023). In Bengalese finches and zebra finches, female-directed song is characterized by an increase in tempo and reduction in the variability of syllable pitch and sequencing (Sossinka and Böhner, 1980; Sakata et al., 2008; Kojima and Doupe, 2011; Aronov and Fee, 2012; Chen et al., 2016; Toccalino et al., 2016; James et al., 2019), consistent with a switch from undirected ‘practice’ song, with high motor variability, to a more stereotyped ‘performance’ version of the song (Jarvis et al., 1998; Singh Alvarado et al., 2021). These differences between female-directed and undirected song, produced in isolation, are mediated by neuromodulatory brain circuits that act on the song production and learning pathways (Jaffe and Brainard, 2020; Singh Alvarado et al., 2021; Ben-Tov et al., 2023; Roeser et al., 2023). The presence of male conspecifics is less studied in these species (Jarvis et al., 1998; Chen et al., 2016).

We found no consistent change in transition entropy related to social context in our data, indicating that syllable sequencing was equally variable in all contexts. This difference to the existing literature may be due to the small sample size in our study, or due to the prolonged co-housing situation. Most prior work on female-directed song comes from extremely short exposures to a female, typically 30 s–2 min (Sakata and Brainard, 2009; Aronov and Fee, 2012; Picardo et al., 2016; Toccalino et al., 2016). The quality and quantity of directed song can vary within interactions with a female, with changes to transition entropy attenuating within the first hour of exposure to a new female (Heinig et al., 2014; Toccalino et al., 2016). Therefore, we might expect that any differences in the MF condition related to female-directed song would be negligible in our dataset, where birds are co-housed continuously over several days. The remaining high sequencing variability could also explain why degree of learning was not reduced as expected in the MF condition, because the performance variability characteristic of undirected song is a crucial substrate for motor learning (Sober and Brainard, 2012; Dhawale et al., 2017; Tachibana et al., 2022b).

### Neuronal mechanism of song modification learning

Learned modifications of adult song rely at least partly on the anterior forebrain pathway (AFP) (Brainard and Doupe, 2000; Andalman and Fee, 2009; Warren et al., 2011; Kawaji et al., 2024), a circuit which is also crucial for imitative song learning in juveniles (Bottjer et al., 1984; Scharff and Nottebohm, 1991; Ölveczky et al., 2005). The AFP contains two parallel loops through basal ganglia, thalamus and cortex (Jarvis et al., 1998). The lateral subdivision is responsible for adaptive modification of syllable pitch, and the mechanism has been investigated in detail (Fee and Goldberg, 2011; Chen and Goldberg, 2020). The medial loop is not well understood but plays a role in controlling syllable sequencing in Bengalese finches (Koparkar et al., 2024).

Within the lateral AFP, the basal ganglia receive information about song performance from a dopaminergic midbrain projection, and these reinforcement signals are necessary and sufficient to drive pitch learning (Gadagkar et al., 2016; Hisey et al., 2018; Xiao et al., 2018; Kearney et al., 2019). The output nucleus of this pathway, LMAN (lateral magnocellular nucleus of the anterior nidopallium) contributes exploratory motor variability and learned pitch bias onto the song motor pathway (Andalman and Fee, 2009; Ali et al., 2013; Tian and Brainard, 2017; McGregor et al., 2022; Tian et al., 2023). The lateral AFP is suppressed during female-directed singing, leading to a reduction in exploratory pitch variability (Kao et al., 2005; Kao and Brainard, 2006; Sakata and Brainard, 2009; Kojima and Doupe, 2011; Woolley et al., 2014) and reversion of recently learned pitch modifications toward baseline (Ali et al., 2013).

The neuronal mechanisms of learned modification of syllable sequencing are not equally well understood (Warren et al., 2012; Veit et al., 2021; Kawaji et al., 2024). It is tempting to speculate that sequence learning is mediated analogously by the medial subdivision of the AFP, since its output nucleus MMAN (medial magnocellular nucleus of the anterior nidopallium) is involved in regulating sequencing variability (Koparkar et al., 2024). It is possible that the medial AFP analogously integrates reinforcement signals in the basal ganglia (Kawaji et al., 2024) and contributes context-dependent exploratory variability and learned sequencing bias onto the song motor pathway via MMAN.

The dopaminergic evaluation of performance via auditory feedback is reduced during female-directed song in favor of social signals (Roeser et al., 2023), making learning by external reinforcement with non-social auditory stimuli unlikely in this brain state (Sakata and Brainard, 2009). Based on the circuit and behavioral differences during female-directed song, we expected to see no learning during the MF condition. Robust learning in our task could be due to habituation to the female (Heinig et al., 2014; Toccalino et al., 2016), minimizing neuronal activity changes in the AFP. Alternatively, the known AFP changes during female-directed song may primarily affect the lateral AFP (Jarvis et al., 1998) and only regulate female-directed changes to pitch variability, not sequencing (Kao and Brainard, 2006; Hampton et al., 2009; Leblois and Perkel, 2012). This raises the question whether sequencing changes during female-directed song additionally involve other circuits (Hampton et al., 2009; Ali et al., 2013; Jaffe and Brainard, 2020), which may not be affected in an analogous way that inhibits learning in the ‘performance’ mode. Facilitation of reinforcement learning during social housing could therefore be specific only to sequence learning. It would be interesting to perform a similar manipulation of social context during pitch learning, where the neuronal circuit mechanisms are better understood.

### Influence of social context on learning

Birdsong learning by social imitation of a live tutor is a well-known example of social learning in animals (Zentall, 2022). In tutoring studies, better learning from interacting with a live tutor (Deregnaucourt et al., 2013; Chen et al., 2016; Tanaka et al., 2018; Carouso-Peck and Goldstein, 2019) can be explained by the live tutor representing a stronger multimodal stimulus for imitation, and by social feedback about song performance from the tutors or other conspecifics (West and King, 1988; Chen et al., 2016; Carouso-Peck and Goldstein, 2019). Social learning has also been demonstrated in finches for tasks other than song learning (Katz and Lachlan, 2003; Guillette and Healy, 2017; Narula et al., 2018; Breen et al., 2019). In contrast, we here report influences of social context on learning in a computer-controlled song modification task, in which the conspecific is not required for learning. These influences may therefore be seen as a form of social facilitation, where the mere presence of another individual can influence attentional or motivational processes which enhance the degree of learning also outside of social imitative learning (Zajonc, 1965; Zentall, 2022). For example, the increase in song speed in the MM and MF conditions could be taken as a sign of increased arousal (Cooper and Goller, 2006; Jaffe and Brainard, 2020), which may make the negative auditory feedback more aversive, and therefore provoke stronger song adjustments per individual feedback experience. Additionally, it is possible that social context indirectly affects the degree of learning via other variables, such as increased movement or preening in social conditions compared to the single-housed condition (Vignal et al., 2004; Aronov and Fee, 2012). For example, the presence and identity of neighboring conspecifics has dramatic effects on behavioral patterns in monkeys (Testard et al., 2024). Finally, signals from the social partner may provide negative or positive social feedback to adjust song away from song renditions that elicit WN, if WN is perceived as unpleasant by the social partner (West and King, 1988; Olsson et al., 2020).

One motivation for this study was to test whether social housing is feasible for behavioral and neuronal studies of sequence learning in Bengalese finches. We demonstrate that sequence learning is possible, and may even be enhanced, for social housing. This degree of learning was achieved with a substantially lower singing rate in the social conditions. However, the dramatically reduced singing rate in both MF and MM conditions (Yamahachi et al., 2017), may make social housing prohibitive for most studies on the neuronal mechanisms of song production. Given the variability in degree of learning and other complexities added by the social context, it may continue to be essential to perform most learning experiments on birds that are housed alone for the duration of the experiment.

## Data Availability

The datasets presented in this study can be found in online repositories. The names of the repository/repositories and accession number(s) can be found below: https://github.com/veitlab/SocialContextLearning.
