# Supplementary material for: Social context affects sequence modification learning in birdsong
Source: Front Psychol. 2025 Feb 5;16:1488762. doi: 10.3389/fpsyg.2025.1488762 (PMC11835814; doi:10.3389/fpsyg.2025.1488762)
Supplement: Supplementary file 1 [file Data_Sheet_1.pdf]

Supplementary Material

Supplementary Figure 1. (A) Distribution of transition entropy per bout for each subject (bin size = 0.05; kernel used for smoothing = 1). Vertical bars indicate mean transition entropy. **(B)** Distribution of sequence durations [ms] as a measure of song speed for each subject (bin size = 5; kernel used for smoothing = 1). Vertical bars indicate mean sequence duration. **(C)** Transition entropy and song speed for each song bout during baseline screening for all birds. Mean values for each social context are depicted in bold. We could not distinguish directed from undirected song in these data, and did not find a pattern of correlation that was consistent across birds or contexts. Only regression lines for statistically significant correlations are shown along with the correlation coefficient R (bird1, $pMF = 0.045$; bird2, $pMF = 0.026$, $pMM = 0.000011$; bird5, $pMM = 0.016$; bird6, $pMF = 0.0053$). Colors indicate social context (MA, male alone; MF, male-female; MM, male-male). Note: only data from bouts which contained the chunk used for determining the song speed were included in the correlation analysis and are shown in this figure.

Supplementary Figure 2. (A) Degree of learning and mean song rate during training (number of bouts per day, measured between 9:30am and 5pm). Degree of learning tended to be higher in the social conditions, which had lower song rate than the MA condition, but there was no significant correlation between song rate and degree of learning on the group level ($p = 0.78$, n = 18). **(B)** Degree of learning and baseline transition probabilities. There was no significant correlation between degree of learning and BS transition probability ($p = 0.057$, n = 18) **(C)** Absolute change in transition probability and BS transition probability. There was no significant correlation between absolute change in transition probability during learning and BS transition probability ($p = 0.055$, n = 18). Colors indicate social context (MA, male alone; MF, male-female; MM, male-male). The individual birds are indicated by different marker styles. BS = baseline screening. The shaded triangle marks impossible reductions in transition probability that would be higher than baseline probability.

Supplementary Figure 3. (A) Hourly song rate [counts] for example bird 1 during baseline recording for all social contexts. **(B)** Maximum song rate for each social context. When comparing only the time windows with the maximum song rate rather than the total song rate (as in Fig. 1C), singing remains suppressed in the social contexts (MF and MM) compared to the single-housed context (MA). One-way ANOVA: $^{**}p = 0.002$; Multiple comparison test: MA-MF, $p = 0.003$; MA-MM, $p = 0.012$.

Supplementary Table 1. Number of sequences that contained the target transition during baseline and on the best training day, compared to the total number of occurrences of the target branch point. All subjects with the exception of bird5_MF significantly reduced the proportion of target transitions at the target branch point in all social contexts (proportion test, p-values in right column).

		Baseline Data			Training Data (T_{best})			
Subject	Social Context	Occurrence of targeted transition (counts)	Occurrence of transitions from branch point syllable (counts)	%	Occurrence of targeted transition (counts)	Occurrence of transitions from branch point syllable (counts)	%	p-values
Bird 1	MA	957	2305	41.5	370	1039	35.6	0.0014
	MF	742	1543	48.1	157	645	24.3	<0.001
	MM	488	1063	45.9	151	857	17.6	<0.001
Bird 2	MA	234	2890	8.1	86	2045	4.2	<0.001
	MF	172	1962	8.8	14	1282	1.1	<0.001
	MM	186	2211	8.4	23	1388	1.7	<0.001
Bird 3	MA	442	531	83.2	828	1258	65.8	<0.001
	MF	584	988	59.1	336	748	44.9	<0.001
	MM	380	621	61.2	220	543	40.5	<0.001
Bird 4	MA	1652	2498	66.1	278	1634	17.0	<0.001
	MF	812	1060	76.6	138	687	20.1	<0.001
	MM	766	1043	73.4	309	538	57.4	<0.001
Bird 5	MA	1603	2438	65.8	370	962	38.5	<0.001
	MF	138	587	23.5	83	385	21.6	0.5
	MM	755	1966	38.4	118	745	15.8	<0.001
Bird 6	MA	1444	2596	55.6	401	1027	39.0	<0.001
	MF	291	333	87.4	286	377	75.9	<0.001
	MM	1178	2093	56.3	497	1841	27.0	<0.001
