# Supplementary figures and images for: Social context affects sequence modification learning in birdsong
Source: Front Psychol. 2025 Feb 5;16:1488762. doi: 10.3389/fpsyg.2025.1488762 (PMC11835814; doi:10.3389/fpsyg.2025.1488762)

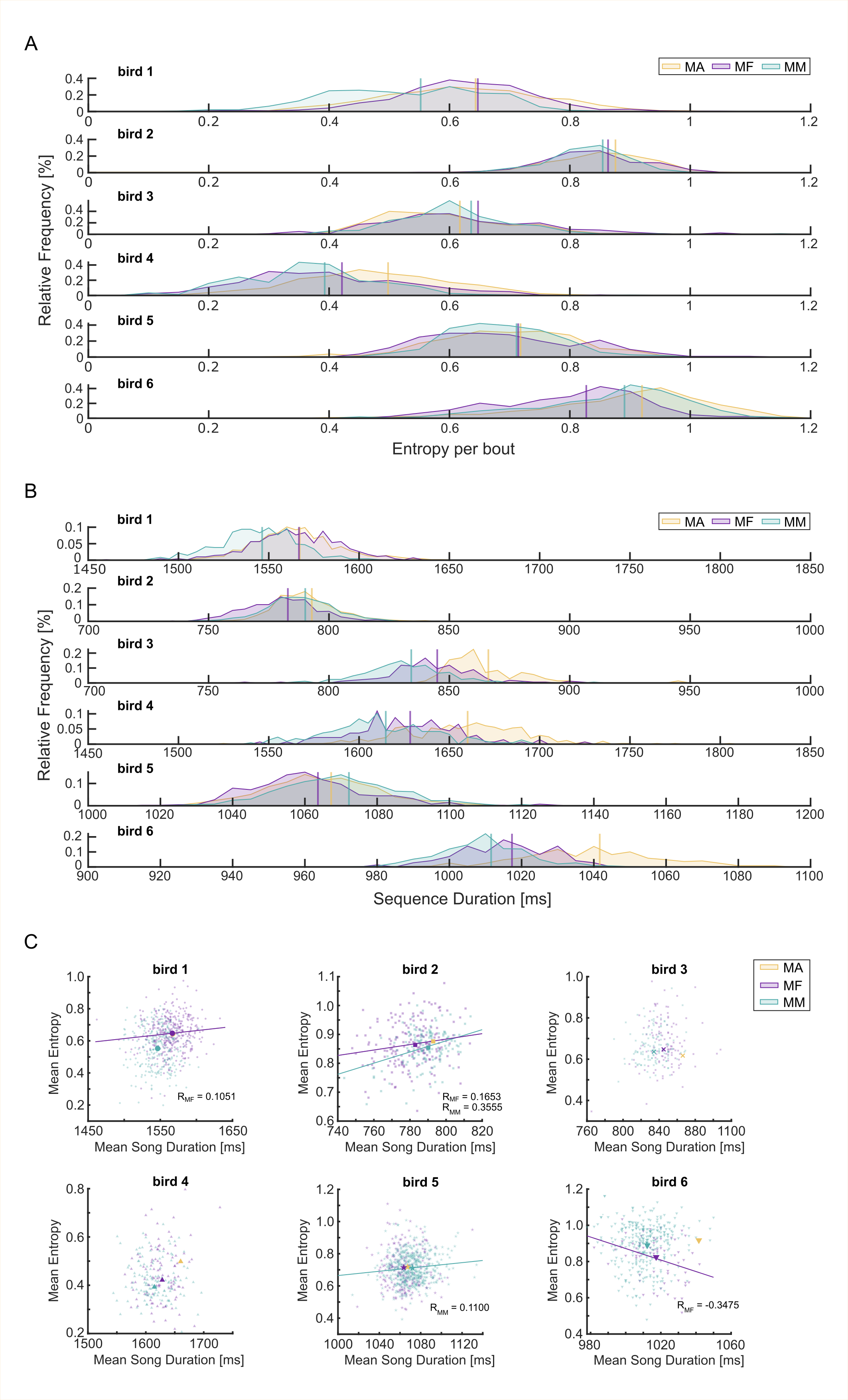

Supplement: Supplementary file 2 [file Image_1.tiff]

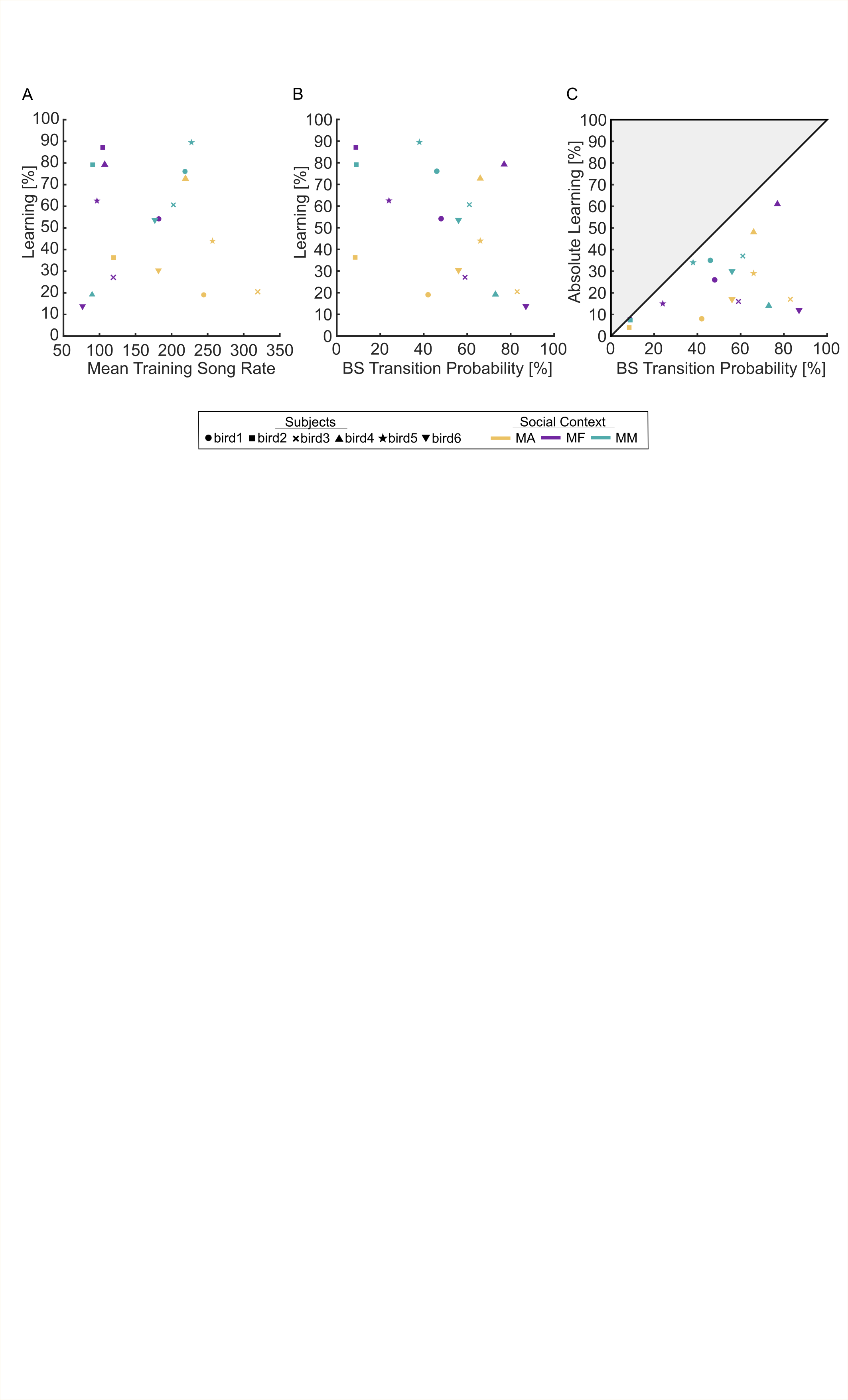

Supplement: Supplementary file 3 [file Image_2.tiff]

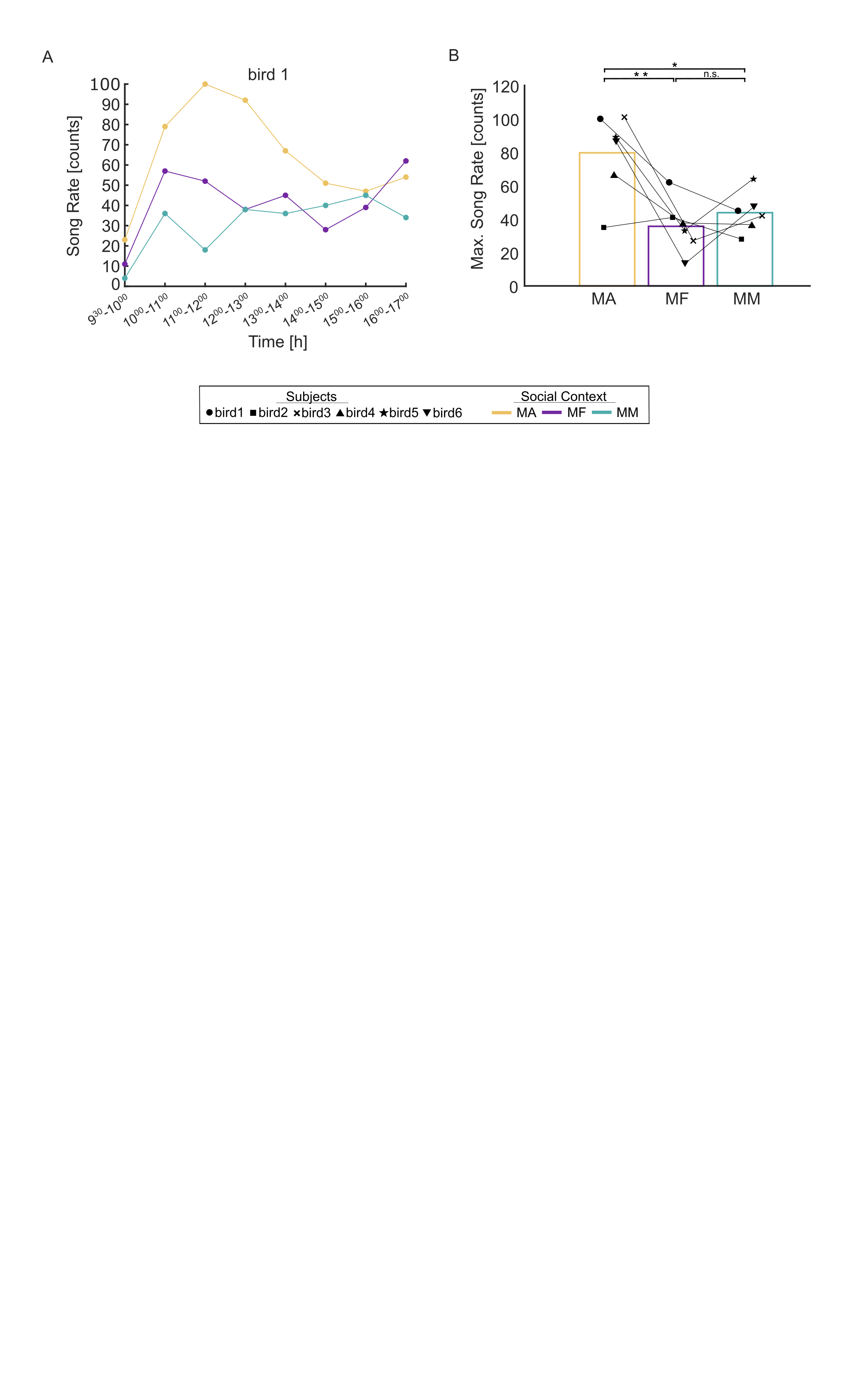

Supplement: Supplementary file 4 [file Image_3.tiff]
